# Impact of body mass index on the early experience of robotic pancreaticoduodenectomy

**DOI:** 10.1007/s13304-021-01065-9

**Published:** 2021-05-19

**Authors:** Ying-Jui Chao, Ting-Kai Liao, Ping-Jui Su, Chih-Jung Wang, Yan-Shen Shan

**Affiliations:** 1 Institute of Clinical Medicine, College of Medicine, National Cheng Kung University, 138, Sheng-Li Road, Tainan, 70428 Taiwan; 2 Department of Surgery, College of Medicine, National Cheng Kung University Hospital, Tainan, Taiwan

**Keywords:** Body mass index, Obesity, Robot, Pancreaticoduodenectomy, Learning curve

## Abstract

Obesity increases surgical morbidity and mortality in open pancreaticoduodenectomy (OPD). Its influence on robotic pancreaticoduodenectomy (RPD) remains uncertain. This study aimed to investigate the impact of body mass index (BMI) on the early experience of RPD. Between June 2015 and April 2020, 68 consecutive RPDs were performed at the National Cheng Kung University Hospital. The patients were categorized as normal-weight (BMI < 23 kg/m^2^), overweight (BMI = 23–27.5 kg/m^2^), and obese (BMI > 27.5 kg/m^2^) according to the definition of obesity in Asian people from the World Health Organization expert consultation. Preoperative characteristics, operative details, and postoperative outcomes were prospectively collected. The cumulative sum was used to assess the learning curves. The average age of the patients was 64.8 ± 11.7 years with an average BMI of 24.6 ± 3.7 kg/m^2^ (23 normal-weight, 29 overweight, and 16 obese patients). Eighteen patients were required to overcome the learning curve. The overall complication rate was 51.5%, and the major complication rate (Clavien grade ≥ III) was 19.1%. The normal-weight group showed the most favorable outcomes. The blood loss, major complication rate, peripancreatic fluid collection rate, and conversion rate were higher in the obese group than in the non-obese group. There were no differences in the operative time, clinically relevant postoperative pancreatic fistula, postoperative hemorrhage, delayed gastric emptying, bile leak, wound infection, reoperation, hospital stay, and readmission rate between the obese and non-obese groups. Multivariate analysis showed obesity as the only independent factor for major complications (OR: 5.983, CI: 1.394–25.682, *p* = 0.001), indicating that obesity should be considered as a surgical risk factor during the implementation of RPD.

**Supplementary Information:**

The online version contains supplementary material available at 10.1007/s13304-021-01065-9.

## Introduction

Obesity is one of the most critical public health problems in the world and the prevalence of obesity has a rapidly increased among the Taiwanese and Chinese populations in recent decades [1, 2]. Obesity is not only associated with an increased risk of developing metabolic syndrome and cardiovascular diseases but has been considered as a surgical risk factor for complications in various kinds of abdominal surgeries. Increased fat deposition in the visceral organs and a heavy omentum would impair surgical exposure and obscure the operative field, resulting in longer operative time and more blood loss. Hence, obesity is considered an independent factor for conversion to open surgery, which might result in a longer operation time, greater blood loss, and higher postoperative complications [3–5].

Pancreaticoduodenectomy (PD) is one of the most complicated operations in abdominal surgeries, requiring extensive dissection and three delicate digestive reconstructions. The mortality rate substantially decreases to 2–5%, and the postoperative morbidity remains as high as 40–60% [6–9]. Previous studies have reported increased morbidity and mortality in obese patients undergoing open pancreaticoduodenectomy (OPD) when compared with non-obese patients [10–13]. The robotic platform provides surgeons with ergonomic conditions and dexterity and has an increasing application in general, urological, and gynecological procedures; however, the penetration of robotic pancreaticoduodenectomy (RPD) is still slow and uncommon owing to the complex high-risk procedure and steep learning curve. RPD has been shown to provide better postoperative outcomes including lesser blood loss, lesser surgical site infection, fewer pulmonary complications, and a shorter hospital stay than OPD [14–16]. Case selection is an important issue in RPDs, especially during the learning curve phase. Although obesity is not a contraindication of minimally invasive pancreatic resection (MIPR) [17], the body mass index (BMI) should be considered when assessing surgical risks. However, the impact of BMI on postoperative morbidities of RPD has seldom been discussed. The purpose of this study was to investigate the impact of BMI on surgical outcomes during the early development period of RPD at a high-volume tertiary hospital.

## Materials and methods

Between June 2015 and April 2020, 68 consecutive RPDs were performed at the National Cheng Kung University Hospital, a tertiary referral center in Tainan, Taiwan. The first RPD was completed in June 2015 after the installation of the da Vinci Si surgical system (Intuitive Surgical Inc., Sunnyvale, CA) and all operations were performed using the da Vinci Si surgical system. The indications for RPD were periampullary tumors and the patients who were generally suitable for laparoscopic surgery; the specific contraindications for RPD included large tumors (> 5 cm), tumors with major vessel invasion, locally advanced tumors, tumors with bulky lymphadenopathy, previous severe pancreatitis, and previous major abdominal surgery. For pancreatic adenocarcinoma, the tumor with a size larger than 2 cm was considered a contraindication in our study due to a high risk of vessel invasion and the concern of tumor dissemination during dissection., BMI > 35 mg/m^2^ was considered as a relative contraindication for RPD. All operations were performed by a single experienced laparoscopic surgeon (YJ Chao) and assisted by senior experienced surgeons with experience in advanced laparoscopic techniques. Before the first RPD, the surgeon had performed 210 OPDs, 72 laparoscopic distal pancreatectomies, 10 robotic distal pancreatectomies, and 8 robotic distal gastrectomies. The patients were divided into three groups: normal weight, BMI < 23 kg/m^2^; overweight; BMI = 23–27.5 kg/m^2^; and obese, BMI > 27.5 kg/m^2^, according to the definition of overweight and obesity in Asian individuals from the World Health Organization expert consultation [18]. The dilated pancreatic duct was defined as a pancreatic duct diameter > 3 mm. All events recorded within 90 days of surgery were reported as postoperative complications. Postoperative complications were stratified according to the Clavien–Dindo classification [19]. Delayed gastric emptying (DGE), postoperative pancreatic fistula (POPF), and postoperative pancreatectomy hemorrhage (PPH) were determined based on the International Study Group of Pancreatic Surgery [20–22]. The conversion was defined as a change from robotic surgery to open surgery. Reoperation was considered a secondary operation because of severe complications after RPD. Re-admission was defined as any surgery-related admission within 30 days after discharge. Mortality was defined as any death occurring within 90 days or during the index hospitalization. Data were collected prospectively from June 2015 to August 2020, including clinicopathological characteristics and postoperative outcomes. This study was approved by the Institutional Review Board of the National Cheng Kung University Hospital and written informed consent was obtained from all patients.

### Surgical procedure

After the induction of general anesthesia, the patient is placed in 30-degree reverse Trendelenburg position with legs split and the table is rotated 10 degrees to the left. Pneumoperitoneum is created from the sub-umbilical port and the pneumoperitoneum pressure is set a routine pressure of 12 mmHg. Laparoscopic exploration is performed to assess unexpected peritoneal seeding or liver metastasis. The remaining 5 trocars are shown in the Supplementary Fig. 1. The robotic system is docked over the patient’s head. The assistant surgeon stands between the patient’s legs. The left lobe of the liver is retracted using the W-shaped liver retraction technique to expose the porta hepatis [23]. The transverse colon is lifted upward to expose the fourth part of the duodenum and the proximal jejunum is subsequently divided at 20 cm distal to the ligament of Treitz. An extensive Kocher maneuver is performed to mobilize the transverse colon and duodenum. The right gastrocolic and right gastric vessels are clipped using Hem-o-lok and divided. The proximal duodenum is divided using a linear stapler. The pylorus and first portion of the duodenum are usually preserved for pylorus-preserving PD if no tumor invasion or obvious duodenal ulcer is observed. The lymph node stations 8a and 12a are harvested and the gastroduodenal artery is clipped using Hem-o-lok and divided. Cholecystectomy is performed and the common bile duct was dissected away from the hepatic artery and portal vein. The retropancreatic tunnel is dissected and the pancreatic neck is transected using ultrasonic shears. The uncinate process is dissected from the right side margin of the superior mesenteric artery, and the common bile duct is subsequently transected. Pancreatic anastomosis is performed using a modified Blumgart anastomosis. An internal stent is routinely placed if the diameter of the pancreatic duct is less than 5 mm. The hepaticojejunostomy is created using a one-layer continuous suture with 4-0 or 5-0 prolene. For pylorus-preserving PD, duodenojejunostomy is performed through mini-laparotomy after specimen retrieval. For classical PD, a linear stapled gastrojejunostomy is created intracorporeally. Before the closure of the abdominal wound, two Jackson Pratt drains are routinely inserted at the Morrison pouch and behind the pancreaticojejunostomy, respectively.

### Statistics

The results were expressed as the mean ± standard variation or median with interquartile range for quantitative data. The Chi-square test, Fisher exact test, one-way analysis of variance among groups, and Kruskal–Wallis test followed by all pairwise comparisons were used for statistical analysis. The learning curve was measured using the cumulative sum analysis (CUSUM) method to assess the technical competence of certain procedures [24]. The CUSUM of the operative time (CUSUM_OT_) was measured from the first to the last patient. The CUSUM_OT_ was calculated as CUSUMOT = ∑^in^ = 1(*x*_*i*_ − μ), where *x*_*i*_ is the mean value of the overall operation time and μ is the operation time of each case. Statistical significance was set at *p* < 0.05. All data analyses were performed using SPSS version 19.0 (IBM SPSS, Chicago, IL, USA).

## Results

The mean age of the patients was 64.8 ± 11.7 years including 30 men and 38 women. The average BMI was 24.6 ± 3.7 kg/m^2^. There were 23 normal-weight, 29 overweight, and 16 obese patients. A total of 18 patients had dilated pancreatic ducts. The mean operative time was 317 ± 67 min and the estimated blood loss was 155 ± 217 mL. Pylorus-preserving PD was performed in 57 patients (83.8%) and traditional PD in 11 patients (16.2%). Pathologic examinations showed ampullary adenocarcinoma (33.8%), pancreatic adenocarcinoma (19.1%), cholangiocarcinoma (13.2%), intraductal papillary mucinous neoplasm (16.2%), ampullary adenoma (5.9%), chronic pancreatitis (2.9%), neuroendocrine tumor (1.5%), and other benign diseases (7.4%). Two patients (2.9%) were converted to open surgery in the obese group due to severe inflammation at the pancreatic head and tumor adhesive to the superior mesenteric vein and none in the normal-weight and overweight groups. Thirty-three patients experienced complications with an overall complication rate of 51.5%, and the major complication rate (Clavien grade ≥ III) was 19.1%. There were 17.6% CR-POPF (16.1% grade B POPF, 1.5% grade C POPF), 8.9% grade B/C PPH, 11.8% grade B/C DGE, and 5.9% bile leakage. Twelve patients (17.6%) had peripancreatic fluid collections, and eight of them required drainage. One patient required reoperation due to failed embolization of the pseudoaneurysm from the gastroduodenal artery due to POPF. Eventually, the patient had multiple organ failure and died on a postoperative day 114. The median hospital stay was 15 days (interquartile range: 11–22 days), with an 11.8% readmission rate (Supplemental Table 1).

### Learning curve analysis

**Fig. 1:**
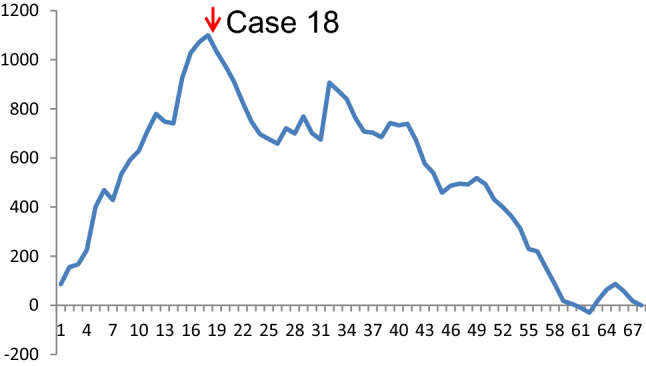
Cumulative sum curve for operative time

**Fig. 2:**
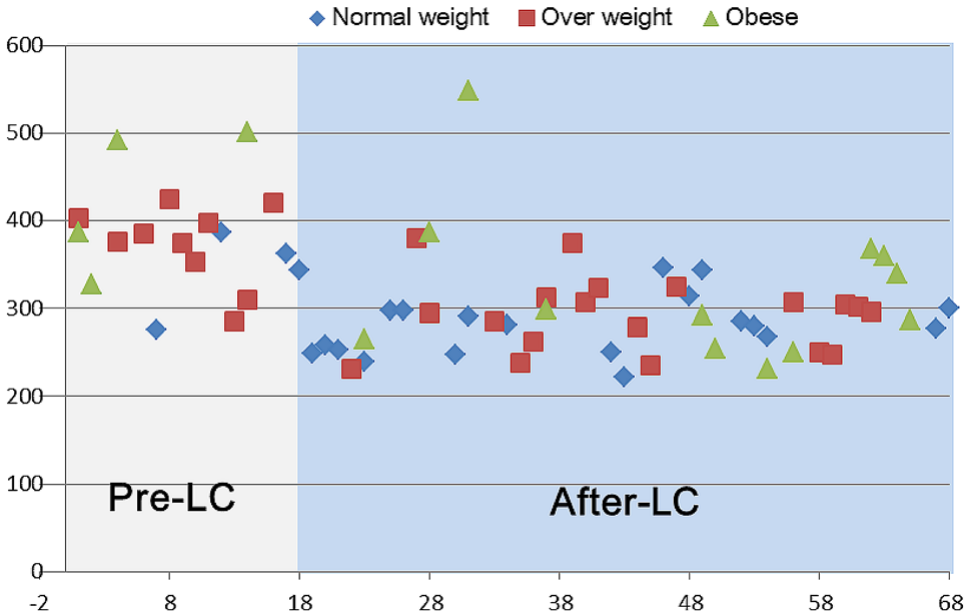
Weight status and operative time among the 68 consecutive patients. *LC* learning curve

The learning curve assessment was performed using the CUSUM method for the operative time. Additionally, the peak indicated the cutoff point of the learning curve, which was observed in the 18th case (Fig. 1). The weight status of the patients (normal-weight, overweight, and obese) was not a contra-indicator for RPD during the pre-learning curve phase and showed a similar distribution between the pre-learning curve and after-learning curve phase (obese/overweight/normal-weight: 4/10/4 in the pre-learning curve phase; 12/19/19 in the post-learning curve phase, *p* = 0.378)(Fig. 2).

### Demographic among the groups of normal weight, overweight, and obese patients

The BMI was significantly different between the three groups (*p* < 0.0001). The operative time was shorter in the normal-weight group than in the overweight (282 vs. 301 min, *p* = 0.049) and obese group (282 vs. 343 min, *p* = 0.043). Meanwhile, the normal-weight group had lower intraoperative blood loss than the normal-weight group (0 vs. 100 mL, *p* = 0.053) and obese groups (0 vs 175 mL, *p* = 0.005). There were no differences in age, sex, ASA score, dilated pancreatic duct, pancreatic texture, operative procedure, pathological diagnosis, distribution of the learning curve, retrieval of lymph nodes, and hospital stay between the groups (Table 1).

**Table 1:** Demographic among normal weight, overweight, and obese groups of patients

	Normal *n* = 23	Overweight *n* = 29	Obese *n* = 16	*p*	P1 normal vs overweight	P2 Overweight vs obese	P3 Normal vs obese
Age, yr, median (IQR)	66 (57–76)	66 (58–74)	62 (54–73)	0.508	1	0.448	0.525
Gender (Female/male)	15/8	12/17	11/5	0.113	0.103	0.120	1
BMI, kg/m^2^, median (IQR)	21.9 (19.8–22.4)	24.3 (23.8–25.5)	28.9 (28.1–30.3)	< 0.0001	< 0.0001	< 0.0001	< 0.0001
ASA				0.197	0.053	0.390	0.636
I	2 (8.7%)	3 (10.3%)	1 (6.3%)				
II	8 (34.8%)	19 (65.5%)	8 (50%)				
III	13 (56.5%)	7 (24.1%)	7 (43.8%)				
Tumor size, cm, median (IQR)	2 (1.5–3.2)	2.5 (1.3–3.6)	1.8 (1.25–2.1)	0.204	0.356	0.021	0.143
Dilated pancreatic duct	8 (34.8%)	7 (24.1%)	3 (18.8%)	0.5	0.540	1	0.471
Pancreas texture				0.725	0.577	1	1
Soft	21 (91.3%)	28 (96.6%)	15 (93.8%)				
Hard	2 (8.7%)	1 (3.4%)	1 (6.2%)				
Operative procedure				0.874	1	1	0.674
Traditional PD	3 (13.0%)	5 (17.2%)	3 (18.8%)				
Pylorus-preserving PD	20 (87.0%)	24 (82.8%)	13 (81.2%)				
Pathological diagnosis				0.129	0.826	0.068	0.062
Ampullary adenocarcinoma	8 (34.8%)	12 (41.4%)	3 (18.8%)				
Pancreatic adenocarcinoma	6 (26.1%)	5 (17.2%)	2 (12.5%)				
Cholangiocarcinoma	2 (8.7%)	3 (10.3%)	4 (25%)				
IPMN	5 (21.7%)	6 (20.7%)	0				
Ampullary adenoma	0	1 (3.4%)	3 (18.8%)				
Chronic pancreatitis	1 (4.3%)	0	1 (6.2%)				
Neuroendocrine tumor	0	0	1 (6.2%)				
Others	1 (4.3%)	2 (6.9%)	2 (12.5%)				
Learning curve phase				0.378	0.217	0.738	0.694
Pre-learning curve	4 (17.4%)	10 (34.4%)	4 (25%)				
After learning curve	19 (82.6%)	19 (65.5%)	12 (75%)				
Operative time, min, median (IQR)	282 (253–314)	307 (282–376)	334 (271–387)	0.065	0.049	0.514	0.043
Blood loss, mL, median (IQR)	0 (0–0)	100 (0–225)	175 (100–588)	0.023	0.053	0.184	0.005
Conversion	0	0	2 (12.5%)	0.053	1	0.121	0.162
Retrieval lymph node number, median (IQR)	13 (10–19)	13.5 (9–19.3)	13.5 (8–21.5)	0.879	0.798	0.903	0.810
Hospital stay	12 (11–16)	16 (11–23)	16 (11–27.8)	0.273	0.16	0.677	0.196

^*^IQR interquartile range

### Morbidity and mortality among groups of normal weight, overweight, and obese patients

The obese group had the highest overall complication rate followed by the overweight and the normal-weight groups (75% vs. 48% vs. 30%), which was significantly higher in the obese group than in the normal weight group (*p* = 0.004) (Table 2). The major complication rate in the obese group was 50%, which was significantly higher than that in the overweight (*p* = 0.037) and normal-weight groups (*p* = 0.001). There was no CR-POPF in the normal-weight group, which was significantly lower than that in the obese group (*p* = 0.033). The obesity group had a higher risk of postoperative peripancreatic fluid collection than the normal-weight group (*p* = 0.019) and overweight group (*p* = 0.021). The PPH, DGE, bile leakage, wound infection, reoperation, and readmission were comparable between the groups.

**Table 2:** Postoperative complications among normal weight, overweight, and obese groups of patients

	Normal *n* = 23	Overweight *n* = 29	Obese *n* = 16	*p*	P1 Normal vs overweight	P2 Overweight vs obese	P3 Normal vs obese
Complication, overall	7 (30.4%)	14 (48.3%)	12 (75%)	0.012	0.149	0.159	0.004
0	16 (69.6%)	15 (51.7%)	4 (25%)				
Grade I	3 (13.0%)	1 (3.4%)	0				
Grade II	4 (17.4%)	8 (27.6%)	4 (25%)				
Grade IIIa	0	3 (10.3%)	6 (37.5%)				
Grade IIIb	0	0	0				
Grade IVa	0	2 (6.9%)	1 (6.25%)				
Grade IVb	0	0	0				
Grade V	0	0	1 (6.25%)				
Major complication, ≧ Grade III	0	5 (17.2%)	8 (50%)	0.002	0.21	0.037	0.001
CR-POPF (Grade B + C)	1 (4.3%)	6 (20.7%)	5 (31.3%)	0.087	0.117	0.483	0.033
Grade B	1 (4.3%)	6 (20.7%)	4 (25%)				
Grade C	0	0	1 (6.3%)				
PPH, Grade B/C	1 (4.3%)	2 (6.9%)	3 (18.8%)	0.264	1	0.330	0.286
DGE, Grade B/C	2 (8.7%)	4 (13.8%)	2 (12.5%)	0.847	0.682	1	1
Bile leakage	0	2 (6.9%)	3 (18.8%)	0.087	0.497	0.330	0.061
Peripancreatic fluid collection	2 (8.7%)	3 (10.3%)	7 (43.8%)	0.007	1	0.021	0.019
Wound infection	3 (13.0%)	3 (10.3%)	3 (18.8%)	0.728	1	0.650	0.674
Reoperation	0	0	1 (6.25%)	0.192	1	0.356	0.410
Readmission	2 (8.7%)	3 (10.3%)	3 (18.8%)	0.601	1	0.650	0.631

^*^CR-POPF clinically relevant postoperative pancreatic fistula; *DGE* delayed gastric emptying; *PPH* postpancreatectomy hemorrhage

We further divided the patients into two groups: non-obese (BMI ≦ 27.5 kg/m^2^) and obese (BMI > 27.5 kg/m^2^). The obese group had more patients with ampullary adenocarcinomas (*p* = 0.011). However, when considering ampullary tumors (adenocarcinomas and adenomas), there was no difference between groups (obese: 44% vs non-obese: 37.5%, *p* = 0.779). Furthermore, the obese group had a smaller tumor size (*p* = 0.031), higher blood loss (*p* = 0.027), and higher conversion rate (11.1% vs. 0%, *p* = 0.053) than those in the non-obese group. There were no significant differences in the age, sex, ASA, dilated pancreatic duct, pancreatic texture, operative procedure, learning curve phase, operative time, retrieval lymph node, and hospital stay (Supplemental Table 2). The obese group had higher overall postoperative complications (75% vs. 40.4%, *p* = 0.005) and major complication rates (50% vs. 9.6%, *p* = 0.001) than the non-obese group (Supplemental Table 3). Finally, we analyzed the risk factors correlated with morbidity during the development period of RPD. In the univariate analysis, obesity and conversion were critical factors leading to major complications. In the multivariate analysis, we found that the only risk factor was obesity (OR [95%CI): 5.983 [1.394–25.682], *p* = 0.016) (Table 3).

**Table 3:** Univariate and multivariate analysis for the risks of major complications after RPD

	Without major complication *n* = 55	Major complication *n* = 13	Univariate model		Multivariate model	
			OR (95% CI)	*p* value	OR (95%CI)	*p* value
Age, median (IQR)	65 (57–76)	65 (59–70)	1.002 (0.951–1.056)	0.939	–	
Gender			0.903 (0.268–3.04)	0.869	–	
Male	24 (43.6%)	6 (46.2%)				
Female	31 (56.4%)	7 (53.8%)				
Obesity status			9.400 (2.448–36.097)	0.001	5.983 (1.394–25.682)	0.016
Non-obese	47 (85.5%)	5 (38.5%)				
Obese	8 (14.5%)	8 (61.5%)				
ASA			1.662 (0.597–4.624)	0.330	–	
I	6 (10.9%)	0				
II	28 (50.9%)	7 (53.8%)				
III	21 (38.2%)	6 (46.2%)				
Tumor size, cm, median (IQR)	2 (1.5–3.2)	1.8 (1–2.3)	0.717 (0.418–1.231)	0.228	–	
Learning curve phase				0.758	–	
Before	15 (27.3%)	3 (23.1%)	1.25 (0.302–5.172)			
After	40 (72.7%)	10 (76.9%)				
Dilated pancreatic duct	16 (29.1%)	2	0.443 (0.088–2.228)	0.323	–	
Pancreas texture			1.202 (0.371–3.889)	0.759	–	
Soft	52 (94.5%)	12 (92.3%)				
Hard	3 (5.5%)	1 (7.7%)				
Operative procedure			1.762 (0.396–7.837)	0.457	–	
Traditional PD	8 (14.5%)	3 (23.1%)				
Pylorus-preserving PD	47 (85.5%)	10 (76.9%)				
Pathological diagnosis			1.063 (0.846–1.337)	0.599	–	
Ampullary adenocarcinoma	19 (34.5%)	4 (30.8%)				
Pancreatic adenocarcinoma	12 (21.8%)	1 (7.7%)				
Cholangiocarcinoma	6 (10.9%)	3 (23.1%)				
IPMN	8 (14.5%)	3 (23.1%)				
Ampullary adenoma	3 (5.5%)	1 (7.7%)				
Chronic pancreatitis	2 (3.6%)	0				
Neuroendocrine tumor	0	1 (7.7%)				
Others	5 (9.1%)	0				
Operative time, min, median (IQR)	298 (259–353) (259–353)	312 (283–395) (283–395)	1.009 (1.000–1.018)	0.048	0.997 (0.981–1.013)	0.693
Blood loss, mL, median (IQR)	100 (0–200)	200 (100–525)	1.004 (1.001–1.007)	0.008	1.003 (0.998–1.008)	0.180

^*^IQR interquartile range

## Discussion

Overweight and obesity have become important health problems in Asian countries as well as in Western countries. Only a few studies from Western countries have focused on the surgical outcomes of PD patients with high BMI, and data with respect to Asians are still limited. This is the first Asian study to investigate the impact of BMI during the implementation of RPD. We stratified the body weight status according to the definition of obesity for the Asian population by the World Health Organization (WHO). We found the obesity group (BMI > 27.5 kg/m^2^) had the highest overall and major complication rate. The major complication rate was significantly higher in the overweight than in the normal-weight and overweight groups. In contrast, the normal-weight group (BMI < 23 kg/m^2^) had the most favorable outcomes, with the lowest overall complication rate and hospital stay. Meanwhile, obesity was the only independent factor for major complications in multivariate analysis. The definition of obesity in Asian population from the World Health Organization expert consultation is feasible to stratify the surgical risks in RPD during the implementation phase. Although obesity is not considered as a contraindication in MIPR according to the Miami International Evidence-based Guideline [17], obesity should be considered as increasing surgical risks and the selection criteria of RPD should be more restricted for obese patients during the implementation phase. Currently, the ideal approach of PD for obese patients remains unclear and only one retrospective study showed that RPD had less wound infection and fewer CR-POPF than OPD [25].

Postoperative peripancreatic fluid collection (43.8%) was the most common complication in obese patients, which might be ascribed to a wider dissection area, larger dead space, more tissue damage, delayed mobilization, and more frequent dysfunction of drains than non-obese patients. The WHO defines overweight as a BMI ≥ 25 kg/m^2^ and obesity as a BMI ≥ 30 kg/m^2^, while the definition of obesity for the Asian is different from that in other countries. Chinese and South Asian individuals usually have a thinner bone and lower muscle mass than Caucasians [26]. Furthermore, Asians have higher body fat and risks of cardiovascular events than Whites for the same body weight and BMI [27]. In particular, a higher body fat contributed to increased abdominal adipose tissue and visceral adipose tissue in Chinese and South Asians [28, 29]. This would increase the surgical challenges and surgical morbidities in digestive surgeries. Previous studies from Western countries have shown that overweight patients receiving OPD may have increased operation time, blood loss [30], and surgical morbidities [10, 12, 13] compared to normal-weight patients. Chang et al. presented data from the National Clinical Database in the United States, which showed that obesity increased the risk of wound infection, reoperation, failure of extubation in 48 h, infection, pulmonary embolism, and renal insufficiency compared to the control group [11]. In an early report from Asia, the Japanese group analyzed 97 patients undergoing OPD and divided them into overweight (BMI > 25 kg/m^2^) and non-overweight groups (BMI ≤ 25 kg/m^2^). They found that the overweight group experienced an increase in the rate of postoperative peripancreatic fluid collection (14.3% vs. 2.9%, *p* < 0.05). Although the categorization of body constitution was not inappropriate for the Asian population, a higher BMI was associated with an increased risk of peripancreatic fluid collection, which is consistent with our results.

Although the first case series of RPD was published in 2003 [31], the application of a robotic system for PD is still slow and uncommon owing to the steep learning curve. Operative time was one of the most commonly used parameters to evaluate the learning curve. A learning curve exists for every procedure and 20–100 cases would be required to overcome the learning curve of RPD from different reports [32–36]. The duration of a learning curve depends on several factors, including the selection of patients, the surgeon’s volume/knowledge/experience for PD, the experience of advanced minimally invasive surgery, and the maturation of the teamwork. In 2015, Boone et al. presented the largest study in the United States, including 200 cases with a mean BMI of 28 ± 5 kg/m^2^ and the learning curve was passed after 80 cases [35] (Table 4). In 2016, Napoli and Boggi et al. analyzed 70 consecutive cases in Italy with a mean BMI of 23.6 kg/m^2^ and found the operative time dropped after 33 cases [32]. Subsequently, the Taiwanese group from Shyr et al. reported that 20 cases were needed to overcome the learning curve [34]. In our series, only 18 cases were required to overcome the learning curve, and the learning curve appeared to decrease with the dissemination of RPD. We had a short learning curve for RPD, which may be due to (1) the previous experience in OPDs and minimally invasive surgery, (2) a matured team of minimally invasive surgery, (3) the well-established procedure that was followed, (4) a relatively lower BMI (24.6 ± 3.7 kg/m^2^) [35]. The appropriate selection of patients during the learning curve phase is the key to decreasing the operative time and blood loss, resulting in the shortening of the learning curve and reducing postoperative morbidities. The learning endpoint by operative time did not reflect the proficiency and the true learning curve might be longer than the CUSUM analysis. In the largest study of RPD from China, 100 patients were required to overcome the learning curve by operative time and the oncological and surgical outcomes were much improved after 250 cases of RPD [36]. They concluded that more than 200 cases were required to measure the learning curve in RPD appropriately. Although we learned how to master the robotic system and passed the learning curve in 18 patients, the proficiency still needs more cases to stabilize the operative time and postoperative morbidity. Hence, the results of this study showed the states during the implementation phase of RPD, and the postoperative outcomes of obesity at the proficiency stage need further investigation with a large patient number.

**Table 4:** The learning curve of RPD from different time period and population

Author	Periods	Year	Case no	BMI, kg/m^2^ mean (SD)	Operative time	CR-POPF rate	Statistic method	The case passing learning curve	Nationality
Boone et al.	2008–2014	2015	200	28 ± 5	483 ± 113	8.5%	CUSUM	80	U.S
Napoli et al.	2008–2014	2016	70	23.6	522 ± 98	16.7%	CUSUM	33	Italy
Shi et al.	2010–2018	2019	450	23.1 ± 3.5	309 ± 87	9.8%	CUSUM	100	China
Zhang et al.	2012–2016	2018	100	23.8 ± 3.6	358 ± 93	11%	CUSUM	40	China
Shyr et al.	2014–2017	2018	61	24 ± 3.7	438 ± 141	22.9%	CUSUM	20	Taiwan
Chao et al.	2015–2020	Current study	68	24.6 ± 3.7	317 ± 67	17.6%	CUSUM	18	Taiwan

Our study had several limitations. First, this was a retrospective study with a selection bias and low number of patients. To avoid early recurrence after RPD, in this study, the number of pancreatic cancers was relatively low, although there was no statistical difference among the disease types. Second, the patients for RPD were well-selected and we avoided difficult cases of RPD even after the learning curve; therefore, we had a low conversion rate (2.9%). We did not use high BMI as an exclusion criterion for RPD initially; the highest BMI was 35.6 kg/m^2^ in our patients. Although BMI > 35 kg/m^2^ was a relative contraindication for RPD in our institute, only three patients (3/311, 1.0%) receiving PD had BMI higher than 35 kg/m^2^ during this period. In Park's study, BMI might not correlate with visceral fat, and high visceral fat was considered a risk factor for developing CR-POPF [37]. However, calculating visceral fat requires additional software, which is inconvenient and unpopular. The application of visceral fat for risk stratification may be limited. Third, our results could not represent other countries in Asia, and some modifications for the definition of obesity should be considered according to different populations.

## Conclusions

During the implementation phase of RPD, obesity is an acceptable risk factor in a well-prepared team and obese patients have a higher major complication rate with more postoperative peripancreatic fluid collection than non-obese patients. Obesity is the only independent factor for major complications in RPD and should be considered when assessing surgical risks during the early development period.

## Supplementary Information

Below is the link to the electronic supplementary material.

Supplementary file1 (DOCX 39 KB)

Supplementary file2 (DOCX 27 KB)
