# Supplementary material for: Impact of body mass index on the early experience of robotic pancreaticoduodenectomy
Source: Updates Surg. 2021 May 19;73(3):929–37. doi: 10.1007/s13304-021-01065-9 (PMC8184700; doi:10.1007/s13304-021-01065-9)
Supplement: Supplementary file 1 — Supplementary file1 (DOCX 39 KB) [file 13304_2021_1065_MOESM1_ESM.docx]

**Figures**

**Fig.1 Cumulative sum curve for operative time.**

**
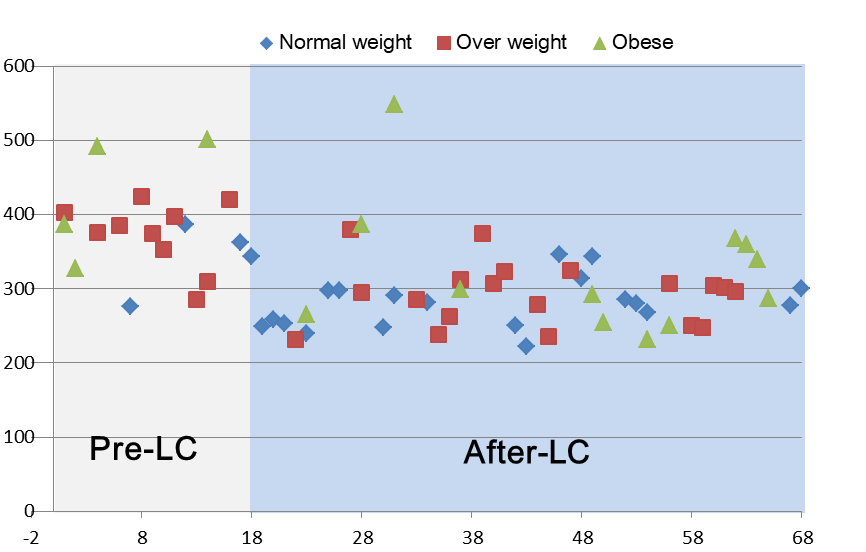
**

**Fig.2 Weight status and operative time among the 68 consecutive patients. LC: learning curve**
