# Supplementary material for: Impact of body mass index on the early experience of robotic pancreaticoduodenectomy
Source: Updates Surg. 2021 May 19;73(3):929–37. doi: 10.1007/s13304-021-01065-9 (PMC8184700; doi:10.1007/s13304-021-01065-9)
Supplement: Supplementary file 2 — Supplementary file2 (DOCX 27 KB) [file 13304_2021_1065_MOESM2_ESM.docx]

| **Supplemental Table1. Patient characteristics and operative results (n=68)** | |
| --- | --- |
| **Age** | 64.8 ± 11.7 |
| **Gender**  **-Male**  **-Female** | 30 (44.1%)  38 (55.9%) |
| **BMI** | 24.6 ± 3.7 |
| **-Normal weight**  **-Overweight**  **-Obese** | 23 (33.8%)  29 (42.6%)  16 (23.5%) |
| **ASA**  **I**  **II**  **III** | 6 (8.8%)  35 (51.5%)  27 (39.7%) |
| **Tumor size, cm** | 2.2 ± 1.3 |
| **Dilated pancreatic duct** | 18 (26.5%) |
| **Pancreas texture**  **-Soft**  **-Hard** | 64 (94.1%)  4 (5.9%) |
| **Operative procedure**  **-Traditional PD**  **-Pylorus-preserving PD** | 11 (16.2%)  57 (83.8%) |
| **Pathological diagnosis**  **-Ampullary adenocarcinoma**  **-Pancreatic adenocarcinoma**  **-Cholangiocarcinoma**  **-IPMN**  **-Ampullary adenoma**  **-Chronic pancreatitis**  **-Neuroendocrine tumor**  **-Others** | 23 (33.8%)  13 (19.1%)  9 (13.2%)  11 (16.2%)  4 (5.9%)  2 (2.9%)  1 (1.5%)  5 (7.4%) |
| **Operative time, min** | 317 ± 67 |
| **Blood loss, ml** | 155 ± 217 |
| **Conversion** | 2 (2.9%) |
| **Retrieval lymph node number** | 14.6 ± 7.1 |
| **Complication (Clavein-Dindo)**  **-0**  **-Grade I**  **-Grade II**  **-Grade IIIa**  **-Grade IIIb**  **-Grade IVa**  **-Grade IVb**  **-Grade V** | 35 (51.5%)  4 (5.9%)  16 (23.5%)  9 (13.2%)  0 (0%)  3 (4.4%)  0 (0%)  1 (1.4%) |
| **CR-POPF, Grade B/ C**  **-Grade B**  **-Grade C** | 12 (17.6%)  11 (16.1%)  1 (1.5%) |
| **PPH, Grade B/C** | 6 (8.9%) |
| **Delayed gastric emptying, Grade B/C** | 8 (11.8%) |
| **Bile leakage** | 4 (5.9%) |
| **Peripancreaic fluid collection** | 12 (17.6%) |
| **Wound infection** | 9 (13.2%) |
| **Reoperation** | 1 (1.5%) |
| **Readmission** | 8 (11.8%) |
| **Hospital stay, median (IQR)** | 15 (11-22) |

**PD: pancreaticoduodenectomy; IPMN: intraductal papillary mucinous neoplasm; CR-POPF: clinically relevant postoperative pancreatic fistula; DGE: delayed gastric emptying; PPH: postpancreatectomy hemorrhage*

**Supplemental Table 2. Demographic between groups of non-obese (normal weight + overweight) and obese patients**

|  | **Non-obese**  **n = 52** | **Obese**  **n = 16** | **P** |
| --- | --- | --- | --- |
| **Age, yr** | 66 (58-75) | 62 (54-73) | 0.426 |
| **Gender (Female/male)** | 27/25 | 11/5 | 0.266 |
| **BMI** | 23.2 (22.1-24.4) | 28.9 (28.1-30.3) | <0.001 |
| **ASA**  **-I**  **-II**  **-III** | 5 (9.6%)  27 (51.9%)  20 (38.7%) | 1 (6.3%)  8 (50%)  7 (43.7%) | 0.882 |
| **Tumor size, cm** | 2.1 (1.4-3.5) | 1.8 (0.85-2.1) | 0.031 |
| **Dilated pancreatic duct** | 15 (28.8%) | 3 (18.8%) | 0.529 |
| **Pancreas texture**  **-Soft**  **-Hard** | 49 (94.2%)  3 (5.8%) | 15 (93.7%)  1 (6.3%) | 1 |
| **Operative procedure**  **-Traditional PD**  **-Pylorus-preserving PD** | 8 (15.4%)  44 (84.6%) | 3 (18.8%)  13 (81.2%) | 0.712 |
| **Pathological diagnosis**  **-Ampullary adenocarcinoma**  **-Pancreatic adenocarcinoma**  **-Cholangiocarcinoma**  **-IPMN**  **-Ampullary adenoma**  **-Chronic pancreatitis**  **-Neuroendocrine tumor**  **-Others** | 20 (38.4%)  11 (21.1%)  5 (9.6%)  11 (21.2%)  3 (5.8%)  1 (1.9%)  0  3 (5.8%) | 3 (18.8%)  2 (12.5%)  4 (25%)  0  3 (18.8%)  1 (6.3%)  1 (6.3%)  2 (12.5%) | 0.011 |
| **Learning curve phase**  **-Pre-learning curve**  **-After learning curve** | 14 (26.9%)  38 (73.1%) | 4 (25%)  12 (75%) | 1 |
| **Operative time, min, median (IQR)** | 298 (264-346) | 334 (271-387) | 0.156 |
| **Blood loss, ml, median (IQR)** | 100 (0-200) | 175 (100-588) | 0.027 |
| **Conversion** | 0 | 2 (12.5%) | 0.053 |
| **Retrieval lymph node, median, (IQR)** | 13 (10-19) | 13.5 (8-21.5) | 0.953 |
| **Hospital stay, day, median (IQR)** | 15 (11-18) | 16 (11-28) | 0.361 |

**Supplemental Table 3. Postoperative complication between groups of non-obese and obese patients.**

|  | **Non-obese**  **n = 52** | **Obese**  **n = 16** | **P** |
| --- | --- | --- | --- |
| **Complication, overall**  **-0**  **-Grade I**  **-Grade II**  **-Grade IIIa**  **-Grade IIIb**  **-Grade IVa**  **-Grade IVb**  **-Grade V** | 21 (40.4%)  31 (59.6%)  4 (7.7%)  12 (23.1%)  3 (5.8%)  0  2 (3.8%)  0  0 | 12 (75%)  4 (25%)  0  4 (25%)  6 (37.5%)  0  1 (6.3%)  0  1 (6.3%) | 0.005 |
| **Major complication,**  **≧ Grade III** | 5 (9.6%) | 8 (50%) | 0.001 |
| **CR-POPF, Grade B+C**  **-Grade B**  **-Grade C** | 7 (13.5%)  7 (13.5%)  0 | 5 (31.3%)  4 (25%)  1 (6.3%) | 0.136 |
| **PPH, Grade B/C** | 3 (5.8%) | 3 (18.8%) | 0.137 |
| **DGE, Grade B/C** | 6 (11.5%) | 2 (12.5%) | 1 |
| **Bile leakage** | 2 (3.8%) | 3 (18.8%) | 0.081 |
| **Peripancreatic Fluid collection** | 5 (9.6%) | 7 (43.8%) | 0.005 |
| **Wound infection** | 6 (11.5%) | 3 (18.8%) | 0.430 |
| **Reoperation** | 0 | 1 (6.3%) | 0.235 |
| **Readmission** | 5 (9.6%) | 3 (18.8%) | 0.380 |

**CR-POPF: clinically relevant postoperative pancreatic fistula; DGE: delayed gastric emptying; PPH: postpancreatectomy hemorrhage*
